# Serum sirtuin 3 levels and multimodal abnormalities in brain structure and function in Parkinson’s disease patients with depression

**DOI:** 10.1007/s10072-025-08170-2

**Published:** 2025-04-21

**Authors:** Zonghan She, Xuelin Qi, Xiaoxue Shi, Haoran Peng, Jinhua Zheng, Jianjun Ma, Yunfei Sun, Mengyan Zhang, Yingyun Wang, Qing Xu, Qi Gu, Siyuan Chen, Xue Li

**Affiliations:** 1 Department of Neurology, Zhengzhou University People’s Hospital, Zhengzhou, China; 2 Department of Neurology, Henan Provincial People’s Hospital, Zhengzhou, China; 3 Department of Neurology, Henan University People’s Hospital, Zhengzhou, China

**Keywords:** Parkinson’s disease, Sirtuin 3, Depression, VBM, FMRI

## Abstract

**Background:**

Depression is a common nonmotor symptom in patients with Parkinson's disease (PD). Currently, few studies have investigated the relationships between serum markers and neuroimaging changes associated with depression in PD patients.

**Objective:**

To explore the correlations among depression, serum SIRT3 levels, and brain structural and functional alterations in PD patients.

**Methods:**

The Hamilton Depression Scale‐17 (HAMD-17) was used to assess depression. Serum SIRT3 levels were measured using an enzyme-linked immunosorbent assay (ELISA). Voxel-based morphometry (VBM) and resting-state functional magnetic resonance imaging (rs-fMRI) were used to examine structural and functional alterations.

**Results:**

Compared to healthy individuals, serum SIRT3 levels were lower in PD patients, especially in those with depression. PD patients with depression had lower total gray matter volume/total intracranial volume (GMV/TIV) ratio, and GMVs of the right amygdala, lower fractional amplitude of low-frequency fluctuations (fALFF) values of the left middle frontal gyrus (*MidFG.L*) and left superior parietal lobule (*SPL.L*), and altered functional connectivity(FC) primarily involving the Salience Network (*SN*) and the default Mode Network (*DMN*) compared to those without depression. Serum SIRT3 levels, total GMV/TIV ratios, and fALFF values of the *MidFG.L* and *SPL.L* have diagnostic value for PD patients with depression, and their combination can improve predictive accuracy.

**Conclusions:**

Depression in PD patients is associated with lower serum SIRT3 levels, right amygdala atrophy, decreased spontaneous activity in *MidFG.L* and *SPL.L*, and altered FC in the *DMN* and *SN*.

## Introduction

Parkinson’s disease (PD) is the most prevalent central nervous system (CNS) movement disorder. It is currently the world’s fastest-growing brain disease [1].Nonmotor symptoms are well known to be a major source of disability for patients with PD and may exceed the burden caused by motor impairment [2].

Depression is one of the most frequently reported neuropsychiatric disturbances in PD patients, which can develop at any stage of PD and, in some cases, precedes the onset of motor symptoms [3, 4]. Recent epidemiologic studies have shown that the prevalence of major depressive disorder in PD patients is about 5–20%, dysthymia 13%, and non-major depressive disorder 10–30%, which means that nearly half of all PD patients will experience depressive symptoms at some point during the course of their illness [5].Studies have demonstrated that depression is associated with poorer prognosis in PD patients, including adverse neurological outcomes, greater disability, accelerated cognitive decline, and increased mortality [6]. Compared with the general population, PD patients with depression are more prone to experiencing anxiety, pessimism, and suicidal ideation without suicidal behaviors, while exhibiting less guilt and self-blame [7]. Additionally, they exhibit higher incidence rates, more complex clinical manifestations, greater treatment non-response, and higher disability, recurrence, and relapse rates. The etiopathophysiology of depression in PD is unclear and remains largely unexplored. Various genetic vulnerabilities, cognitive predispositions, and neurobiological and psychological factors may contribute to the development of depressive-like disorders in PD patients [8, 9]. For example, from a genetic perspective, a study found that the occurrence of PD patients with major depressive disorder can be attributed to AQP9, SPI1, and RPH3 A genes [10]. Cognitively, PD patients with depression are associated with extensive cognitive impairment [11]. Biologically, depression in PD may be associated with dysfunction in the mesolimbic dopaminergic pathway (ventral tegmental area-nucleus accumbens), cortico-striato-thalamic circuits, and brainstem monoaminergic nuclei (locus coeruleus/dorsal raphe) [5]. And from a psychology perspective, the impact of a PD diagnosis can lead to increased risk of psychological distress and depression [12]. Because of the broad spectrum of depressive features and the overlap of symptoms between depression and PD, PD with depression is often underdiagnosed or misdiagnosed, and therefore the search for diagnostic markers specific to PD with depression is essential [13].

Sirtuin 3 (SIRT3) is an NAD^+^-dependent protein deacetylase located within mitochondria that is highly expressed in the brain [14] and plays a role in regulating various cellular processes, including energy metabolism, mitochondrial biogenesis, and protection against oxidative stress [15]. In Parkinson's disease, SIRT3 plays a positive neuroprotective role [16]. Overexpression of SIRT3 not only inhibits α-synuclein (α-syn) aggregation [17], but also reduces nigrostriatal dopaminergic neuronal mortality through various mechanisms such as inhibition of apoptosis and enhancement of mitosis [16, 18]. Meanwhile, the pathogenesis of depression is also related to SIRT3, which can activate the differentiation potential of aged neural stem cells and safeguard neurogenesis in aging and depression [19], and also improve depressive-like behaviors in rat by enhancing mitochondrial energy metabolism, decreasing the level of ROS, and suppressing neuroinflammation [20, 21]. Research has reported decreased SIRT3 levels in the substantia nigra (SNc) and hippocampus in PD patients [22], and similar SIRT3 under-expression has been observed in the hippocampus of depressed rats [20]. Studies have shown that PD with depression may also involve mitochondrial damage, oxidative stress, and neuroinflammation [13]. Therefore, investigating alterations in SIRT3 levels in PD patients with depression represents a significant research endeavor. To the best of our knowledge, no research has investigated the clinical relevance of circulating SIRT3 levels in PD patients with depression.

Neurobiological factors provide a basis for the development of depression in patients with PD [23]. Magnetic resonance imaging (MRI) technology provides insights into abnormal brain function and structure. Voxel-based morphometry (VBM) is a widely used magnetic resonance imaging (MRI)-based method for structural brain analysis [24]. Previous studies have shown significant gray matter (GM) volume/cortical thickness abnormalities in PD patients with depression, including frontal, temporal, precuneus, and cingulate gyri, as well as in the hippocampus, parahippocampal cortices, and amygdala [25]. Resting-state functional magnetic resonance imaging (rs-fMRI) measures spontaneous fluctuations in the blood oxygen level-dependent (BOLD) signal, occurring simultaneously in different brain regions without the subject performing an explicit task [26]. Previous fMRI studies revealed that the prefrontal lobe, limbic system, and basal ganglia are key nodes that are altered in PD patients with depression [23]. For instance, one study found that depression severity in PD patients positively correlated with functional connectivity (FC) between the orbitofrontal cortex, hippocampal complex, cingulate cortex, caudate, and thalamus [27]. Another study demonstrated significantly increased FC between the left ventral caudate and the left middle occipital gyrus in dPD patients compared to ndPD patients or healthy controls [28]. Other studies identified strong correlations between both the amplitude of low-frequency fluctuations (ALFF) in the left median cingulate cortex (*MCC*) and FC linking the left MCC to default mode network (*DMN*) nodes, with depression severity in PD [29]. Collectively, these findings suggest that functional dysfunction in the limbic network, *DMN*, sensorimotor network, and salience network (*SN*) may critically contribute to depression in PD.

Combined analysis of structural and functional imaging, in contrast to single-mode approaches, facilitate the discovery and identification of unique neural patterns in PD patients depression. The integration of serological and imaging markers presents a viable approach for identifying diagnostic markers in the early and accurate detection of depression in PD patients.

Based on this, we investigated the changes in serum SIRT3 levels in PD patients with depression, as well as the particular brain structural and functional characteristics of PD patients with depression using multimodal MRI technology. Our goal was to uncover better objective signs to aid in the early diagnosis of depression in Parkinson's disease patients.

## Materials and methods

### Participants

A total of 138 clinically diagnosed PD patients, 30 depressive disorders patients without PD (DD-PD^−^) and 80 healthy volunteers (HC) were recruited from 2021 to 2025. The inclusion criteria for PD patients were as follows: 1. Patients who met the criteria for clinically definitive diagnostic PD of MDS [30]; 2. Right-handed individuals; 3. There was no use of antidepressants within 12 h prior to the assessment. The exclusion criteria for PD patients were as follows: 1. Patients diagnosed with Parkinsonism-plus syndrome (PPS), such as multiple system atrophy (MSA) or progressive supranuclear palsy (PSP), or those with secondary parkinsonism due to cerebrovascular disease, poisoning, encephalitis, trauma, or drugs; 2. Individuals with other psychiatric disorders, including bipolar disorder or schizophrenia, or a family history of mental illness; 3. Presence of severe systemic diseases, organic brain diseases, brain tumors, history of brain trauma, deep brain stimulation surgery, or other neurosurgical procedures; 4. History of alcohol abuse, drug addiction, or substance misuse; 5. Cognitive impairment as defined by Mini-Mental State Examination (MMSE) scores (illiterate: MMSE < 17; 1–6 years of education: MMSE < 20; 7 or more years of education: MMSE < 24). Inclusion criteria for HC: 1. Age- and gender-matched to PD patients. 2.HAMD- 17 score < 10. Inclusion criteria for DD-PD^-^: 1. Age- and gender- matched to PD patients. 2.No history of PD. 3. HAMD- 17 score ≥ 10. Exclusion criteria for HC and DD-PD^-^were the same as those for PD patients.

### Clinical data

The general data for the selected PD patients, including age, sex, disease duration, and levodopa equivalent daily dose (LEDD), were recorded and counted. The severity of the disease was evaluated using the modified Hoehn and Yahr (H&Y) staging scale and the UPDRS. Motor symptoms were assessed using Part III of the Unified Parkinson’s Disease Rating Scale (UPDRS-III). Nonmotor symptoms were assessed using the Non-Motor Symptom Scale (NMSS). Patient quality of life was evaluated with the 39-item PD Questionnaire (PDQ- 39). The MMSE was used to assess cognitive function.

The HAMD‐17 scale was used to assess the participants'depressive symptoms. The HAMD- 17 is one of the most widely used outcome measures for depression [31]. Previous research has demonstrated that a cutoff score of 9/10 offers high sensitivity and specificity in detecting depression in PD patients [32]. Accordingly, PD patients were categorized into the PD with depression group (HAMD- 17 scores ≥ 10, dPD) and the PD without depression group (HAMD- 17 scores < 10, ndPD).

All assessments were completed once during a patient’s"on"period. Medical history inquiries, physical examinations, and scale scoring were performed by trained neurologists.

### Blood sample collection and measurement of serum SIRT3 Level

Peripheral blood was collected from each subject in test tubes without anticoagulant between 07:30 and 08:30 AM, after an overnight fast, and before breakfast. The sample was allowed to coagulate at room temperature for 30 min, then centrifuged (1000 × g, 15 min). The serum was removed and stored at − 80 °C until measurement. All samples were collected during the patients’ “on” period.

Serum SIRT3 levels were measured using an enzyme-linked immunosorbent assay (ELISA) kit (CUSABIO, China). The detection range of this kit is 15.6–1000 pg/ml, with a sensitivity of 3.9 pg/ml. The intra- and inter-detection variability ranges are < 8% and < 10%, respectively. Biological replicates were analyzed on the same plate according to the manufacturer’s instructions.

### Structural MRI

#### MRI acquisition

Image acquisition was performed using a Siemens MAGNETOM Prisma 3 T MRI scanner with a 64-channel head coil. The T1-weighted 3D-MPRAGE sequence employed the following parameters: Flip angle: 4°, repetition time (TR): 5000 ms, echo time (TE): 3.43 ms, inversion time (T1): 755 ms, slice thickness: 1.00 mm, slice number: 208, bandwidth: 240 Hz/pixel, voxel dimensions: 1.0 × 1.0 × 1.0 mm^3^, matrix size: 256 × 256, field of view: 256 × 256 mm^2^.

#### Structural MRI processing and analysis

MRI scans were visually inspected to eliminate those with significant vascular lesions, space-occupying lesions, or motion artifacts. Statistical Parametric Mapping 8 software (SPM8, Wellcome Trust Centre for Neuroimaging, UCL, London, UK) was used for image preprocessing and VBM data analysis. Initially, unified segmentation was performed on the structural T1-weighted images, followed by spatial normalization of the resulting probability maps for gray matter and white matter to the Montreal Neurological Institute (MNI) template utilizing a sophisticated nonlinear warping algorithm based on diffeomorphic anatomical registration through the exponentiated Lie algebra (DARTEL) technique [33]. The modulated volumes were smoothed with a Gaussian kernel with a full width at half-maximum of 8 mm. Furthermore, values were obtained for total gray matter volume (GMV), total white matter volume (WMV), and cerebrospinal fluid volume (CSFV). The total intracranial volume (TIV) was computed using the formula: TIV = total GMV + total WMV + CSFV.

We utilized SPM8 to conduct VBM on the individually smoothed GMV to assess differences between Parkinson's disease patients with and without depression. TIV, age, and sex were included as covariates to eliminate variations in GMV due to differences in TIV, age, and sex. The significance threshold was defined as a uncorrected *p* = 0.00 l (*p-unc* = 0.001) at the initial voxel level and a family wise error (*FWE*)-corrected *p* = 0.05 (*p-FWE* = 0.05) at the cluster level. False Discovery Rate (*FDR*)-corrected peak level results (*p-FDR*) are also reported. We utilized the brain region exhibiting a significant disparity between the two groups as the region of interest (ROI) and extracted its volume via the DPABI toolbox.

### Resting-state fMRI

#### Resting-state fMRI acquisition

Participants were asked to keep their eyes closed, stay awake, and not think. Functional images were acquired utilizing axial echo-planar imaging with the following parameters: TR = 2,000 ms, TE = 35 ms, flip angle = 80°, FOV = 240 × 240 mm^2^, matrix size = 94 × 94, voxel dimensions = 2.20 × 2.20 × 2.20 mm^3^, slice thickness = 2.2 mm, number of slices = 75, and number of time points = 180.

#### Resting-state fMRI processing and analysis

SPM12b and the Functional Connectivity Toolbox (CONN) (version 22_a [34],) were used for image preprocessing and analysis of the resting-state fMRI data. The steps for preprocessing and denoising are as follows: data were removed for the first 10 time points; time correction; head motion correction; functional outlier detection; functional segmentation and MNI space standardization; structural segmentation and MNI space normalization; functional smoothing; low-frequency drift and high-frequency physiological noise were removed by bandpass filtering (0.01 < frequency < 0.08 Hz) and noise reduction treatment. Detection of image quality to eliminate unwarranted images (head motion translation > 2.5 mm or rotation > 2.5°).

First-level analysis of the CONN pipeline was conducted to generate individual fractional amplitude of low-frequency fluctuations (fALFF) maps to evaluate regional neural activity. Group-level analyses were performed using a general linear model (GLM) to identify brain regions with significant differences in fALFF between the two groups. The results were thresholded using a combination of a cluster-forming *p* < 0.001 voxel-level threshold and a familywise corrected *p-FDR* < 0.05 cluster-size threshold. Next, we extracted the fALFF values in the differential brain regions.

First-level analysis of ROI-to-ROI connectivity (RRC) matrices was estimated to characterize the functional connectivity between each pair of regions among 132 regions comprising 91 cortical and 15 subcortical ROIs from the FSL Harvard–Oxford Atlas [35]. Potential correlations in resting-state activity were explored by applying a general linear model and performing bivariate correlation analysis based on first-level analysis of the CONN pipeline. Group-level analyses were performed using a GLM to analyze functional connectivity differences between the two groups, and each differential connectivity value was extracted. The results were thresholded using a combination of a p < 0.05 connection-level threshold and a familywise corrected p-FDR < 0.05 cluster-level threshold [36].

### Statistical analysis

The normality of distributions was assessed using the *Shapiro–Wilk* (SW) normality test. Continuous data with a normal distribution are presented as the means ± standard deviations (SDs) and were compared between groups using *t* tests (two groups). Data that did not conform to a normal distribution are presented as medians (25 th–75 th percentiles) and were compared between groups using the *Mann–Whitney U* test (two groups) or the *Kruskal–Wallis H* test (three or more groups, post hoc pairwise comparisons were performed using the *Bonferroni* method). Nominal data are presented as percentages, and group comparisons were performed using the *χ2* test. Because not all variables were normally distributed, we chose *Spearman* correlation analysis to assess the correlation between the indicators. To eliminate confounding factors, partial correlation analysis was used to examine the relationships between serum SIRT3 levels, imaging indices, and HAMD scores. All statistical analyses were performed with SPSS version 25.0 (IBM Corporation, Armonk, NY, United States) and GraphPad Prism 8 (GraphPad Software, Inc., San Diego, CA, USA). A p value < 0.05 was considered statistically significant.

## Results

### Clinical and demographic data

#### Demographic and clinical characteristics of the subjects measuring serum SIRT3 levels

Serum SIRT3 levels were measured in 80 PD patients,30 DD-PD^−^, and 80 HCs. Among the 80 PD patients, 43 patients (53.7%) had HAMD scores suggestive of depression. There were no significant differences in age or sex among the dPD, ndPD, DD-PD^−^, and HC groups (all *p* > 0.05). No significant differences were observed in the MMSE, PSQI, NMSS, or PDQ- 13 scores, or LEDD scores between the dPD and the ndPD groups (all *p* > 0.05). However, there were significant differences in the UPDRS-III score, HAMA- 14 score, and PSQI score between the dPD and ndPD groups. (*T* = 2.152, *p* = 0.034; *Z* = 4.131, *p* = 0.000; *Z* = 2.065, *p* = 0.039, respectively) (Table 1).

**Table 1:** Demographic and clinical characteristics of subjects measuring serum SIRT3 levels

Characteristics	ndPD (*n* = 37)	dPD (*n* = 43)	HC	DD-PD^-^	T/Z/H/F/χ2	*p*-value
Age (years)	60.78 ± 6.90	64.23 ± 8.48	62.6 ± 8.04	60.17 ± 8.29	7.031	0.071
Male (%)	26.00(70.20%)	27.00(62.70%)	44.00(55.00%)	15.00 (50.00%)	3.730	0.292
disease duration (yeaes)	3.00(1.75–5.00)	3.50(2.00–6.25)	NA	NA	− 1.264	0.206
age of onset (years)	59.00(52.50–65.00)	60.00(54.00–67.00)	NA	NA	− 1.415	0.157
H-Y stage	2.00(1.25–3.00)	2.50(2.00–3.00)	NA	NA	− 1.250	0.211
LEDD (mg)	500.00(375.00–762.50)	450.00(37.50–750.00)	NA	NA	− 1.349	0.177
UPDRS-III score	27.32 ± 11.73	33.98 ± 15.327	NA	NA	2.152	0.034
HAMD- 17 scores	5.00(3.00–7.00)	15.00(13.00–19.00)	NA	15.27 ± 3.81	1.000	< 0.001^a,b,c^
HAMA- 14 score	15.00(11.00–17.00)	19.00(16.00–22.50)	NA	NA	− 4.131	< 0.001
MMSE score	25.00(23.50–27.50)	25.00(23.00, 27.00)	NA	NA	− 0.580	0.562
PSQI score	6.00(3.50–10.50)	9.00(5.00–14.00)	NA	NA	− 2.065	0.039
NMSS score	25.00(5.50–56.50)	28.00(15.00–45.00)	NA	NA	− 0.082	0.935
PDQ- 39 score	14.00(6.50–27.00)	20.00(9.00–43.00)	NA	NA	− 1.564	0.118

^a^dPD vs. ndPD: *Z* = 7.932, adjusted *p* < 0.001

^b^DD-PD^−^ vs. ndPD: *Z* = 6.726, adjusted *p* < 0.001

^c^ndPD vs. DD-PD^−^: *Z* = 0.593, adjusted *p* = 1.000

#### Demographic and clinical characteristics of the population collecting MRI data

MRI was performed in all the PD patients, 78 (56.5%) of whom had depression. No significant differences in sex, age of onset, LEDD, UPDRS-III score, MMSE score, or NMSS score were observed between the two groups (all *p* > 0.05). However, significant differences in age, disease duration, H-Y stage, HAMA- 14 score, PSQI score, and PDQ- 13 score were observed (*T* = 2.239 *p* = 0.027; *Z* = 2.942 *p* = 0.003; *Z* = 2.158 *p* = 0.031; *Z* = 4.729 *p* = 0.000; *Z* = 2,677 *p* = 0.007; *Z* = 2.539 *p* = 0.011, respectively). (Table 2).

**Table 2:** Demographic and clinical characteristics of subjects for MRI acquisition

Characteristics	dPD (*n* = 78)	ndPD (*n* = 60)	T/*Z*/χ2	*p*-value
Age(years)	64.13 ± 9.06	60.93 ± 7.21	2.239	0.027
Male(%)	50.00(64.10%)	38.00(63.30%)	0.999	0.926
disease duration(yeaes)	4.00(2.00–7.00)	3.00(0.83–4.00)	− 2.942	0.003
age of onset(years)	58.6 ± 12.23	57.62 ± 7.27	0.553	0.581
H-Y stage	2.00(2.00–3.00)	2.00(1.88–2.50)	− 2.158	0.031
LEDD (mg)	432.67 ± 330.91	399.54 ± 305.68	0.609	0.544
UPDRS-III score	36.05 ± 15.80	31.58 ± 14.08	1.726	0.087
HAMD- 17 scores	15.00(12.00–18.00)	5.00(3.00–7.00)	− 10.065	< 0.001
HAMA- 14 score	20.00(16.75–23.00)	15.00(11.25–19.00)	− 4.729	< 0.001
MMSE score	25.00(24.00, 27.00)	25.00(23.50, 28.00)	− 0.370	0.543
PSQI score	9.00(6.00–14.00)	6.00(4.00–10.00)	− 2.677	0.007
NMSS score	27.00(14.75–41.25)	17.50(6.00–39.25)	− 1.8	0.072
PDQ- 39 score	25.00(12.50–42.25)	14.00(7.00–29.25)	− 2.539	0.011

### Serum SIRT3 level and Its correlation with PD and depression

#### Comparison of serum SIRT3 levels among the different groups

**Fig. 1:**
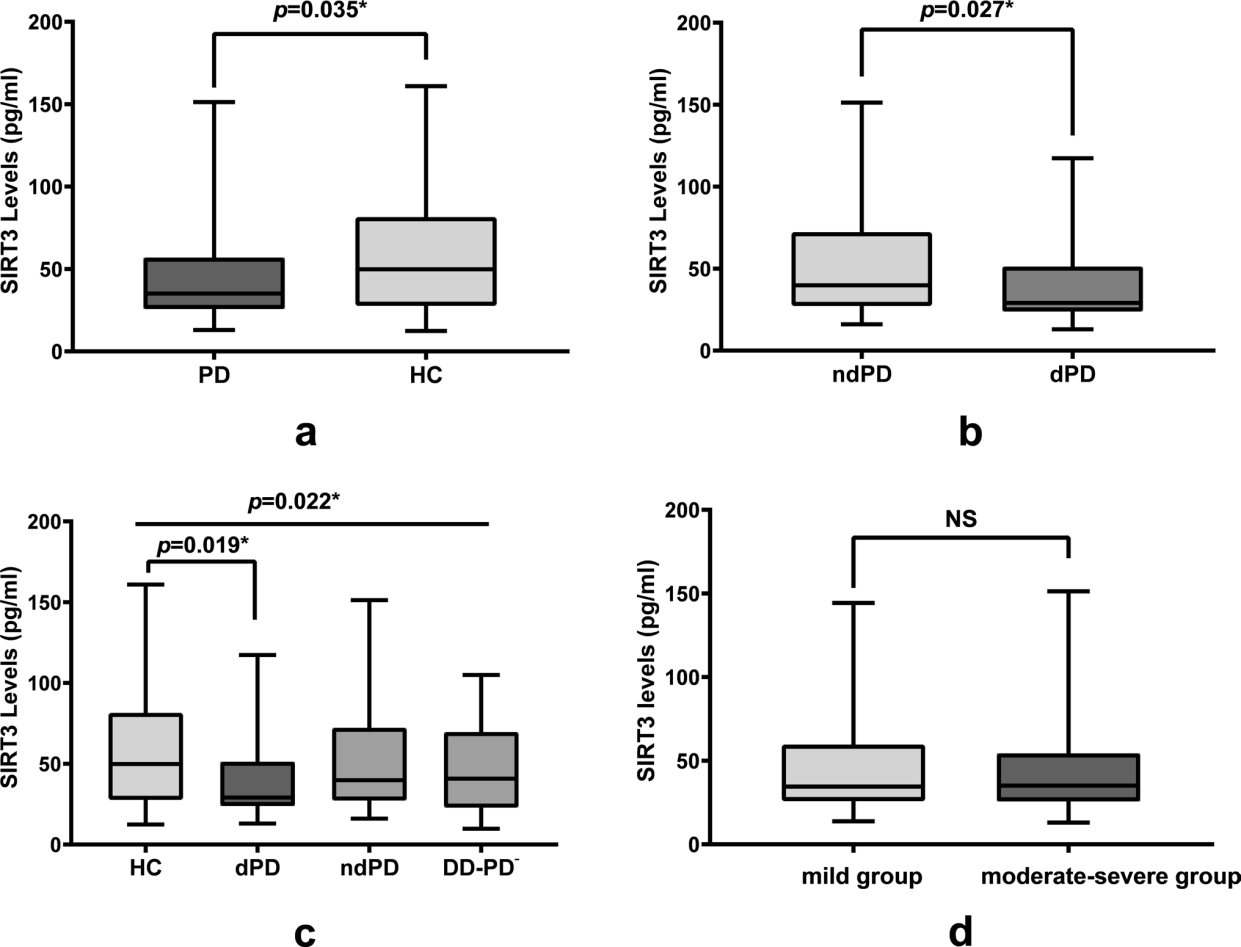
Comparison of SIRT3 levels among different groups. **a** Comparison of SIRT3 levels between PD group and health control (HC) group. **b** Comparison of SIRT3 levels between PD patients with depression (dPD) group and PD patients without depression(ndPD) group. **c** Comparison of SIRT3 levels among depressive disorder patients without PD (DD-PD^−^) group, HC group, dPD group and ndPD group. **d** Comparison of SIRT3 levels between mild group and moderate-severe group

The serum SIRT3 levels in PD patients were significantly lower than those in HCs (35.10 (25.78–56.98) vs. 49.82 (27.82–80.43) ng/mL, *Z* = 2.109, *p* = 0.035) (Fig. 1a). Serum SIRT3 levels were significantly lower in dPD group than in the ndPD group [9.09 (23.81,51.29) vs. 39.87 (27.23,72.28) pg/ml, *Z* = − 2.215, *p* = 0.027] (Fig. 1b).

Serum SIRT3 levels showed statistically significant differences across the dPD, ndPD, HC, and DD-PD^−^groups [29.09(23.81,51.29) vs. 39.87(27.23,72.28) vs. 49.82(27.68,81.54) vs. 40.77(22.84,69.67) pg/ml, *H* = 9.624, *p* = 0.022]. Post hoc pairwise comparisons with Bonferroni correction demonstrated that only the dPD group exhibited significantly lower SIRT3 levels compared to the HC group (*Z* = − 2.949, adjusted *p* = 0.019). No other pairwise comparisons reached statistical significance either after adjustment (all adjusted *p* > 0.05) (Fig. 1c).

Based on the UPDRS-III severity, PD patients were categorized into mild group (UPDRS-III < 32, n = 45) and moderate-severe group (UPDRS-III ≥ 32, n = 35), and no significant SIRT3 differences were found between the two groups [33.616(25.780, 56.976) vs. 36.068(25.630, 57.363) pg/ml, *Z* = − 0.254, *p* = 0.723] (Fig. 1d).

#### Correlations among serum biomarkers, depression scale scores, and clinical features in PD patients

**Fig. 2:**
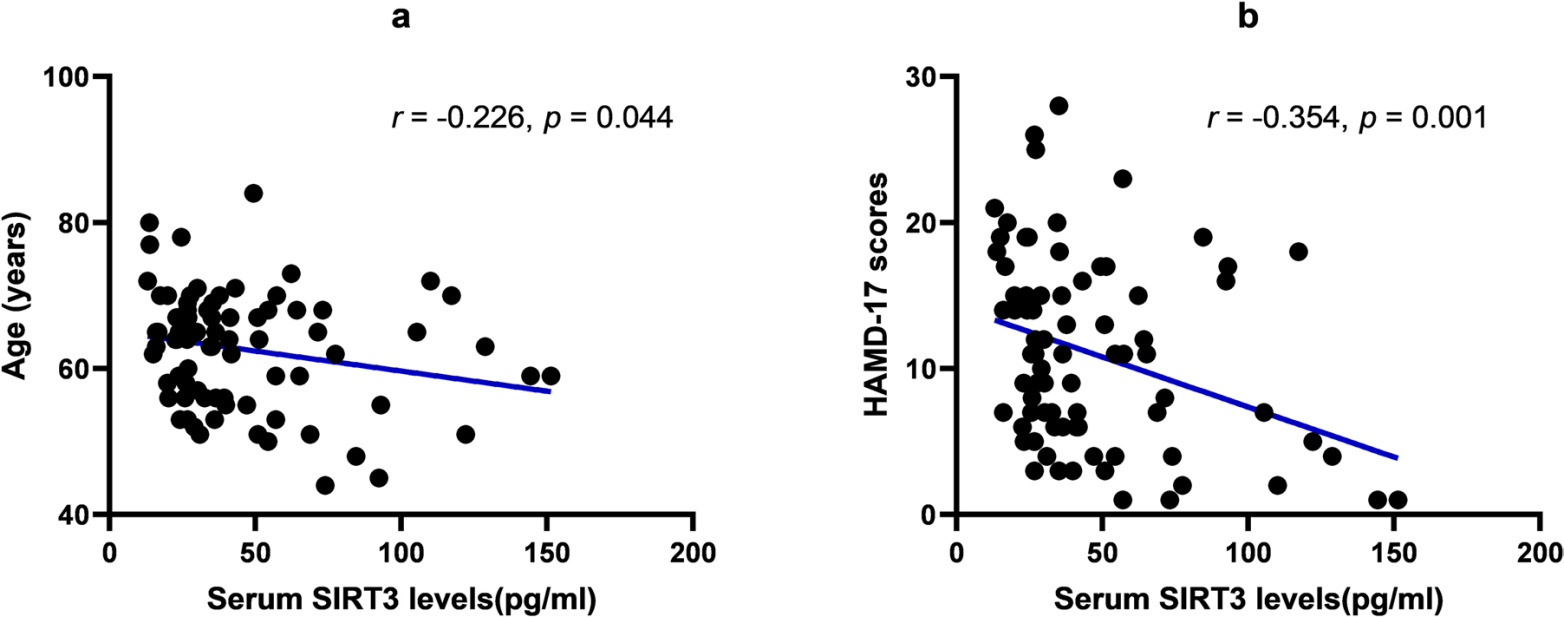
Correlation of serum SIRT3 levels with age and depression scores. **a** Correlation of serum SIRT3 levels with age; **b** Correlation of serum SIRT3 levels with HAMD- 17 scores

The SIRT3 level was negatively correlated with age (*r* = − 0.226, *p* = 0.044) and the HAMD score (*r* = − 0.354, *p* = 0.001) (Fig. 2). No relationships were detected between SIRT3 levels and age of onset, disease duration, LEDD, H-Y stage, UPDRS-III, HAMA, MMSE, PSQI, NMSS, or PDQ- 39 scores (*p* > 0.05).

After adjusting for age, disease duration, and LEDD, multivariate regression analysis revealed no significant association between serum SIRT3 levels and UPDRS-III scores (*β* = 0.006, *t* = 0.049, *p* = 0.961). In contrast, serum SIRT3 levels exhibited a significant negative association with HAMD- 17 scores (*B* = − 0.062, *SE* = 0.022, *β* = − 0.3, *t* = − 2.841, *p* = 0.006, 95% CI: − 0.106 to − 0.019).

Partial correlation analysis revealed that SIRT3 levels were negatively correlated with HAMD scores after adjusting for age, disease duration, H-Y stage, HAMA scores, and LEDD (*r* = − 0.349, *p* = 0.002)..

### Structural MRI results

#### VBM results in PD patients with and without depression

The results indicated that the total GMV/TIV ratio was significantly lower in the dPD group than in the ndPD group (*Z* = − 3.638, *p* = 0.000). In contrast, the difference in the total WMV/TIV ratio was not significant (*T* = 1.613, *p* = 0.109).

**Fig. 3:**
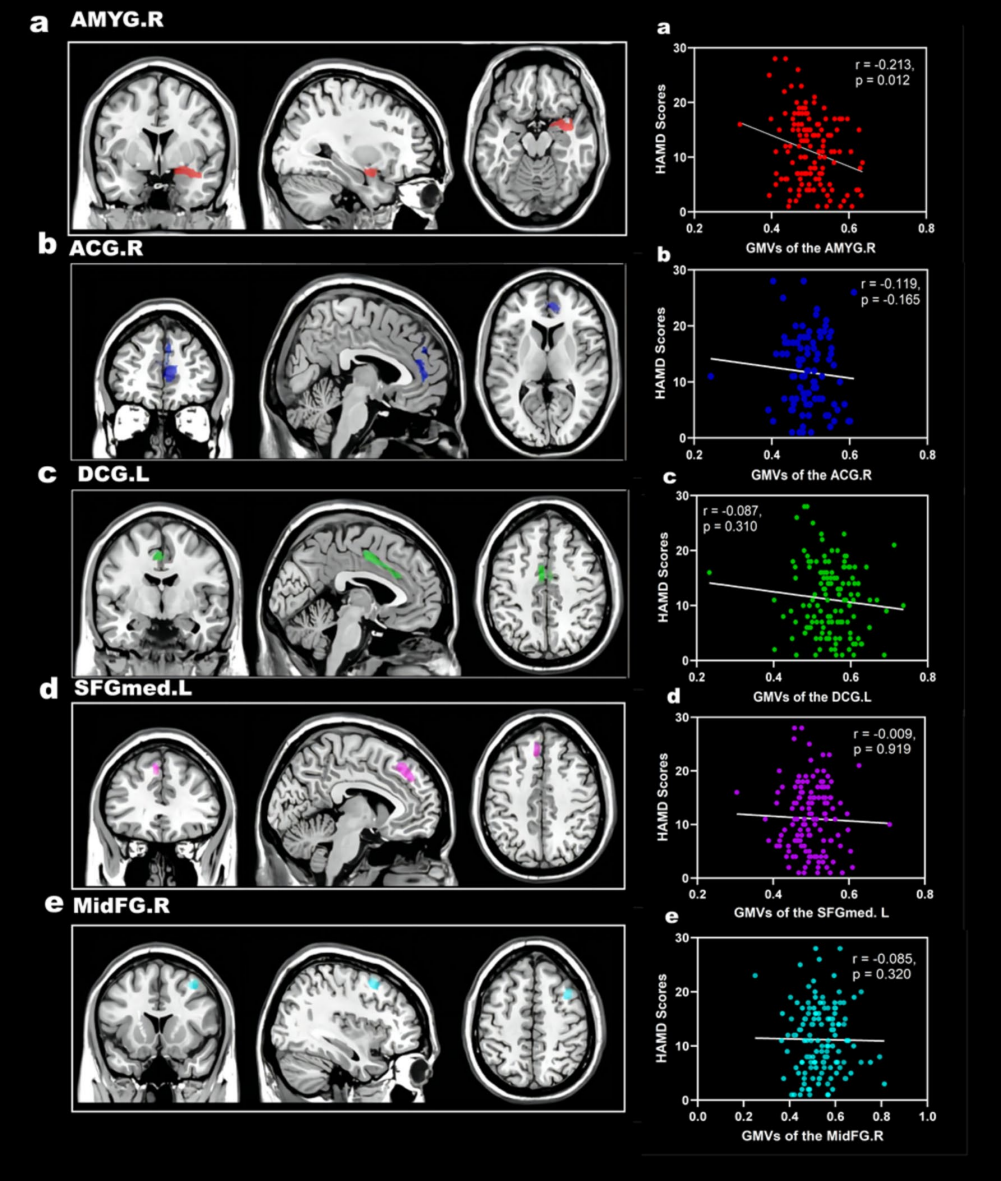
Clusters of significant cortical atrophy in PD patients with depression compared with those without depression (initial voxel level *p-unc* < 0.001and cluster size > 300 voxels) and the results of *Spearman* correlation analysis between the atrophy cortical volume and HAMD-17 scores. Abbreviations: PD, Parkinson's disease; *AMYG*, amygdala; *ACG*, cingulum_Ant, anterior cingulate and paracingulate gyri; *DCG*, median cingulate and paracingulate gyri; *SFGmed*, superior frontal gyrus, medial*; MidFG*, middle frontal gyrus

Compared with the ndPD group, the dPD group presented smaller GMVs in the right amygdala (*AMYG.R*), right anterior cingulate and paracingulate gyri (*ACG.R*), left median cingulate and paracingulate gyri (*DCG.L*), left superior frontal gyrus, medial (*SFGmed. L*) and right middle frontal gyrus (*MidFG.R*) with a initial voxel level *p-unc* < 0.001, and a cluster size > 300 voxels. DPD patients presented markedly reduced GMVs in the *AMYG.R* with a initial voxel level *p-unc* < 0.001and a cluster level *p-FWE* < 0.05. (Table 3, Fig. 3).

**Table 3:** VBM showing areas of gray matter atrophy in PD patients with depression relative to PD patients without depression

Region	R/L	Cluster size	MNI coordinates			*T*	Voxel-level	Peak-level	Cluster-level
			X	Y	Z	value	p (uncorrected)	p (FDR-corr)	p (FWE-corr)
*AMYG*	R	867	37.5	12	− 21	4.0972	< 0.001	0.0207	0.0320
*ACG*	R	673	4.5	46.5	6	4.5089	< 0.001	0.0207	0.0578
*DCG*	L	568	− 3	− 7.5	46.5	4.3163	< 0.001	0.0207	0.0812
*SFGmed*	L	318	− 6	33	45	4.0084	< 0.001	0.0207	0.1958
*MidFG*	R	308	34.5	21	46.5	3.9667	< 0.001	0.0207	0.2033

*AMYG* amygdala, *ACG* anterior cingulate and paracingulate gyri, *DCG* median cingulate and paracingulate gyri, *SFGmed* superior frontal gyrus, medial*, MidFG* middle frontal gyrus

#### Correlations between differential brain regions and HAMD scores in PD patients

We extracted the volumes of five brain regions, *AMYG.R*, *ACG.R*, *DCG.L*, *SFGmed.L*, and *MidFG.R*. These regional GMVs, along with the total GMV/TIV ratios, will be incorporated into correlation analyses with HAMD- 17 scores. The results revealed that the total GMV/TIV ratio and the volume of the *AMYG.R* cortex were negatively correlated with HAMD scores (*r* = − 0.348, *p* = 0.000; *r* = − 0.213, *p* = 0.012, respectively). However, there was no meaningful correlation between the volume of the *ACG.R*, *DCG.L*, *SFGmed.L*, and *MidFG.R* cortices and HAMD scores (r = − 0.119, *p* = − 0.165; r = − 0.087, *p* = 0.310, *r* = − 0.009, *p* = 0.919; *r* = − 0.085, *p* = 0.320, respectively) (Fig. 3).

After adjusting for age, disease duration, H-Y stage, HAMA scores, and LEDD, partial correlation analysis revealed that the total GMV/TIV ratio and the GMVs of the *AMYG.R* were also negatively correlated with HAMD scores (*r* = − 0.354, *p* = 0.000;* r* = − 0.264, *p* = 0.002, respectively).

### RS-fMRI analysis results

#### FALFF Results in PD patients with and without depression

**Fig. 4:**
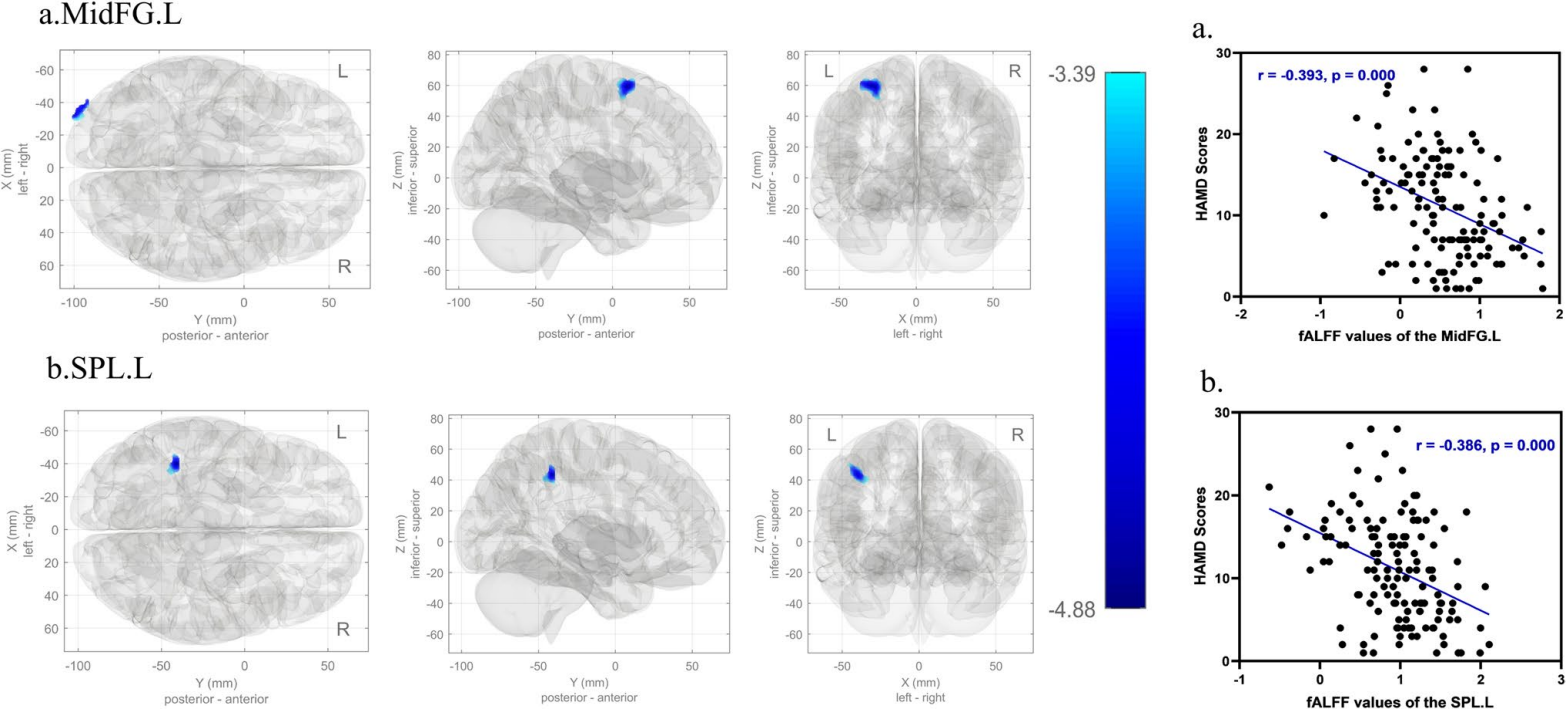
FALFF values in PD patients with depression compared to without, and results of *Spearman* correlation analysis between the fALFF values in the abnormal brain regions and HAMD scores. Abbreviations: PD, Parkinson's disease; fALFF, the fractional amplitude of low frequency fluctuations; *MidFG.L,* left Middle Frontal Gyrus; *SPL.L,* Left Superior Parietal Lobule

FALFF values in the left middle frontal gyrus (*MidFG.L*) and left superior parietal lobule (*SPL.L*) were significantly lower in the dPD group than in the ndPD group (*p-FDR* < 0.05) (Fig. 4).

The fALFF values of the *MidFG.L* and *SPL.L* were negatively correlated with the HAMD score (*r* = − 0.393, *p* = 0.000; *r* = − 0.386, *p* = 0.000, respectively) (Fig. 4).

Partial correlation analysis with age, disease duration, H-Y stage, HAMA scores, and LEDD as confounders revealed the fALFF values of the *MiFG.L* and *SPL.L* were negatively correlated with the HAMD scores (*r* = − 0.325, *p* = 0.000; *r* = − 0.288, *p* = 0.001, respectively).

#### Comparison of ROI-to-ROI functional connectivity between PD patients with and without depression

**Fig. 5:**
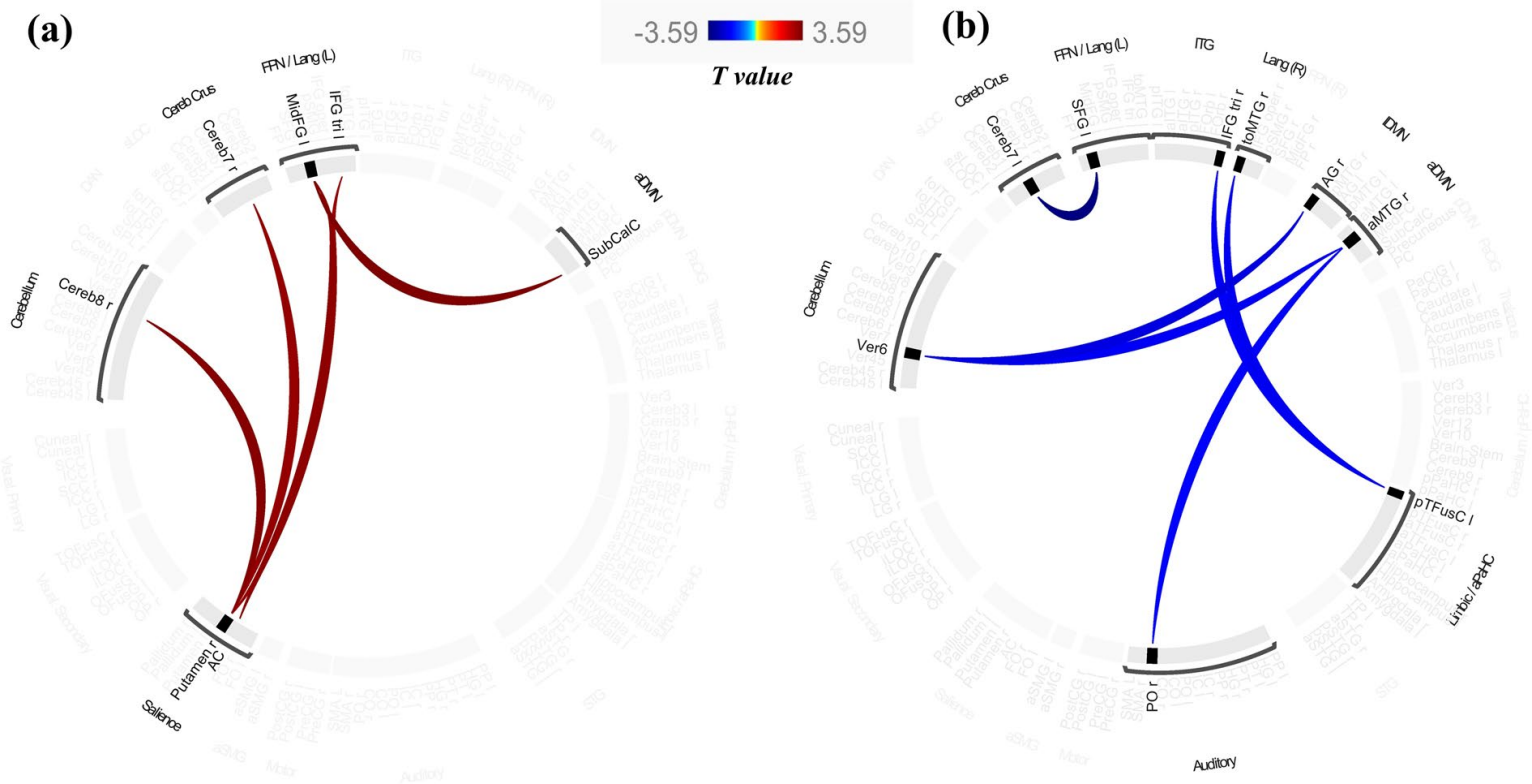
Differences in functional connectivity between patients with Parkinson’s disease with and without depression. **a** Higher connectivity in PD patients with depression compared to those without. **b** Lower connectivity in PD patients with depression compared to those without. *Abbreviations*: *MidFG*, middle frontal gyrus; *SubCalC*, subcallosal cortex; *AC*, cingulate gyrus, anterior division; *Cereb8*, cerebelum 8; *Cereb7*, cerebelum 7; *IFG tri*, inferior frontal gyrus, pars triangularis; *Ver6*, vermis 6; AG, angular gyrus; *aMTG*, right middle temporal gyrus, anterior division; *pTFusC*, temporal fusiform cortex, posterior division; *toMTG*, middle temporal gyrus, temporooccipital part; *SFG*, left superior frontal gyrus; *PO*, parietal operculum cortex; PD, Parkinson’s disease; *R*, right; *L*, left

Compared to ndPD patients, dPD patients had significantly higher functional connectivity (FC) between various brain regions as follows: (1) between the left middle frontal gyrus (*MidFG.L)* and the subcallosal cortex (*SubCalC),* and the the cingulate gyrus, anterior division (*AC)* (all *p-FDR* < 0.05); (2) between the right putamen (*Putamen.R)* and the right cerebelum 8 (*Cereb8.R)*, the right cerebelum 7b (*Cereb7.R)*, and the left inferior frontal gyrus, pars triangularis (*IFG tri.L)* (all *p-FDR* < 0.05). Conversely, dPD patients showed significantly lower functional connectivity between the following region: (1) between the vermis 6 (*Ver6*) and the right angular gyrus (*AG.R*), and the right middle temporal gyrus, anterior division (*aMTG.R*) (all *p-FDR* < 0.05); (2) between the left temporal fusiform cortex, posterior division (*pTFusC.L*) and the right inferior frontal gyrus, pars triangularis (*IFG tri.R*), and the right middle temporal gyrus, temporooccipital part (*toMTG.R*) (all *p-FDR* < 0.05); (3) between the left cerebelum 7b (*Cereb7.L*) and the left superior frontal gyrus (*SFG.L*) (*p-FDR* < 0.05); (4) between the *aMTG.R* and the right parietal operculum cortex (*PO.R*) (*p-FDR* < 0.05).(Table 4)(Fig. 5).

**Table 4:** Differences in functional connectivity between patients with Parkinson’s disease with and without depression

Connection	*T* value	*p-FDR*
Higher connectivity in PD patients with depression		
MidFG.L—SubCalC	3.4	0.001656
MidFG.L- AC	3.3	0.001656
Putamen.R—Cereb8.R	3.36	0.001656
Putamen.R—Cereb7.R	3.25	0.001656
Putamen.R—IFG tri.L	3.21	0.001656
Lower connectivity in PD patients with depression		
Ver6—AG.R	− 3.53	0.001301
Ver6—aMTG.R	− 3.34	0.001311
pTFusC.L—IFG tri.R	− 3.32	0.001311
pTFusC.L—toMTG.R	− 3.38	0.001311
Cereb7.L—SFG.L	− 4.37	0.000112
aMTG.R—PO.R	− 3.23	0.001549

*MidFG* middle frontal gyrus, *SubCalC* subcallosal cortex, *AC* cingulate gyrus, anterior division, *Cereb8* cerebelum 8, *Cereb7* cerebelum 7, *IFG tri* inferior frontal gyrus, pars triangularis, *Ver6* vermis 6, *AG* angular gyrus, *aMTG* right middle temporal gyrus, anterior division, *pTFusC* temporal fusiform cortex, posterior division, *toMTG* middle temporal gyrus, temporooccipital part, *SFG* left superior frontal gyrus, *PO* parietal operculum cortex, *FDR* false discovery rate, *PD* Parkinson’s disease, *R* right, *L* left

### Correlation between serum SIRT3 levels and imaging markers

The results of partial correlation analysis with age, sex, H-Y stage, and disease duration as control factors revealed no significant correlation between serum SIRT3 levels and the total GMV/TIV ratio or GMVs of the *AMYG.R*, GMVs of the *ACG.R*, GMVs of the *DCG.L*, GMVs of the *SFGmed.L*, GMVs of the *MidFG.R*, fALFF values of the *MidFG.L* and fALFF values of the *SPL.L* (all *p* > 0.05).

### Analysis of Serum SIRT3 and imaging markers for the prediction of PD with depression

According to the ROC curve analysis, the area under the curve (AUC) for PD depression based on serum SIRT3 levels was 0.644 (*p* < 0.05, 95% CI: 0.524–0.764), with a sensitivity of 53.5%, specificity of 46.5%, and a cutoff value of 30.042 pg/ml. The AUC for dPD patients based on the *AMYG.R* cortex was 0.570 (*p* = 0.158, 95% CI: 0.473–0.668), because *p* > 0.05, indicating the volume of the AMYG. R cortex has no value in diagnosing PD with depression. The AUC for dPD patients based on the total GMV/TIV ratio was 0.681 (*p* < 0.05, 95% CI: 0.591–0.771), with a sensitivity of 62.80%, specificity of 68.30%, and a cutoff value of 0.422. AUC for dPD patients based on fALFF values in the *MidFG.L* and *SPL.L* were 0.771 (*p* < 0.05, 95% CI: 0.692–0.850) and 0.730 (p < 0.05, 95% CI: 0.647–0.814), respectively. The sensitivity was 68.8% and 87.0%, and specificity was 76.7% and 48.3%, respectively. The optimal cutoff values were 0.510 and 1.232. (Fig. 5).

**Fig. 6:**
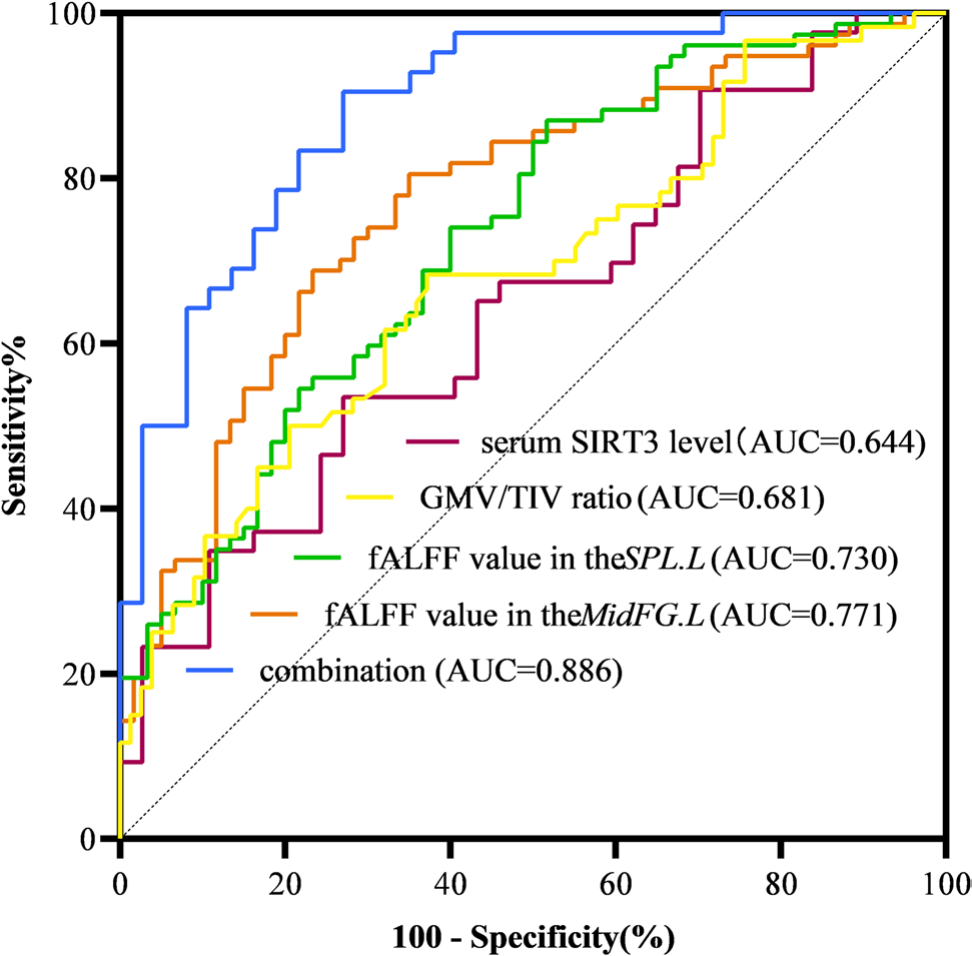
ROC curve of serum SIRT3 level, total GMV/TIV ratios, fALFF value in the *MidFG.L*, fALFF value in the SPL.L, and the combination for the diagnosis of PD with depression. Abbreviations: ROC, receiver operating characteristic; PD, Parkinson's disease; SIRT3, sirtuin3; fALFF, the fractional amplitude of low frequency fluctuations; *MidFG.L,* left middle frontal gyrus; *SPL.L,* left superior parietal lobule

The ROC curve for the combination of serum SIRT3 levels, total GMV/TIV ratios, and fALFF values in the *MidFG.L,* and fALFF values in the *SPL.L* had an AUC of 0.886 (*p* < 0.05, 95% CI: 0.814–0.958), a sensitivity of 90.5%, and specificity of 73.0%. The AUC, sensitivity, and specificity of the combination prediction were better than those of the serum SIRT3 level, the total GMV/TIV ratio, and the fALFF value of the *SPL.L* alone. Compared with the fALFF values of the *MidFG.L* alone, the AUC and sensitivity of the joint prediction are improved, but the specificity is reduced (Fig. 6).

## Discussion

This study examined changes in serum SIRT3 levels and used multimodal MRI to analyze the structural and functional brain characteristics of PD patients with depression to provide objective indicators for early diagnosis. The results indicate that serum SIRT3 levels were significantly lower in PD patients compared to healthy controls, with a more pronounced difference observed in those with depression. In comparison to ndPD patients, dPD patients exhibited significantly reduced total GMV/TIV ratios, GMVs of *AMYG.R*, and fALFF values of *MidFG.L* and *SPL.L*, which correlated with the severity of depression. In comparison to ndPD patients, dPD patients exhibit significant FC abnormalities, particularly within the Salience Network (*SN*) and the Default Mode Network (*DMN*). Serum SIRT3 levels, total GMV/TIV ratios, and fALFF values of the *MidFG.L* and *SPL.L* demonstrate diagnostic utility for PD patients with depression, and their integration enhances diagnostic accuracy.

This study found that reduced SIRT3 levels were exclusively observed in dPD patients, whereas no reductions were detected in the ndPD patients and DD-PD^−^ patients. These results suggest that SIRT3 abnormalities are not a direct consequence of PD-related neurodegeneration but rather a unique pathological feature of PD with depression, and implying distinct underlying mechanisms of dPD compared to primary depression. Previous research has demonstrated that SIRT3 plays a protective role in both PD and depression by maintaining mitochondrial homeostasis, reducing oxidative stress, and inhibiting neuroinflammation [16, 17, 19–21, 37]. The lower SIRT3 levels in dPD patients may indicate that mitochondrial dysfunction, oxidative stress, and neuroinflammation are also involved in the pathogenesis of dPD, aligning with prior findings [13]. Additionally, the reduced SIRT3 levels might reflect that the presence of depression impairs SIRT3-mediated neuroprotection in PD. However, these hypotheses remain preliminary, as this cross-sectional study cannot establish causality or mechanistic insights between SIRT3 reduction and depression in PD. Further longitudinal studies or interventional trials are required to validate these relationships.

Furthermore, we observed that SIRT3 levels were not significantly associated with motor symptom severity in PD patients but showed a significant negative correlation with depression severity, and SIRT3 levels demonstrated predictive value for dPD patients. These findings suggest that reduced SIRT3 levels are not a passive reflection of disease progression but are linked to depression, supporting SIRT3 as an objective diagnostic biomarker for identifying dPD patients. Additionally, the study found a negative correlation between serum SIRT3 levels and age in PD patients, which may be attributed to age-related declines in NAD^+^ (a critical cofactor for SIRT3 activity) [38]. Notably, even in age-matched cohorts, SIRT3 levels in dPD patients remained significantly lower than those in HC, further highlighting depression as the central factor influencing SIRT3 levels.

In conclusion, this study identified serum SIRT3 levels as a potential diagnostic biomarker for dPD for the first time and provided preliminary evidence of SIRT3’s protective role in this population. Future research should focus on unraveling the mechanistic role of SIRT3 in dPD.

The amygdala is an evolutionarily conserved core structure in emotion processing and a key region in emotion and clinical neuroscience [39]. Studies have shown that Lewy lesions in the amygdala begin to appear in PD patients at Braak stage 3 [40]. Previous multiple-image studies have demonstrated that structural damage in the amygdala plays an important role in PD depression. For example, Surdhar et al. [41]proposed a link between the bilateral amygdala and depressive symptoms in PD patients, similar to those in primary major depression. However, van Mierlo et al. [42] said that only the GMVs of the *AMYG.R* was negatively linked to depression scores in PD patients, which matches what we found. In addition, a role for the right amygdala in the pathophysiology of depression in PD is also supported by PET imaging using a marker for dopamine and the noradrenaline transporter: ligand binding was found to be significantly reduced in the right amygdala [43]. The structural damage in the amygdala in PD patients may be due to the deposition of Lewy bodies and Lewy neurites in the amygdala, resulting in the loss of glutamatergic neurons and GABAergic (GMAB) neurons.

The *MidFG* is a region of the dorsolateral prefrontal cortex (DLPFC), is responsible for the top-down regulation of emotional processing and numerous cognitive functions, has a strong correlation with depression [44, 45]. The DLPFC has been identified as a crucial target for depression treatment using repetitive transcranial magnetic stimulation (rTMS) [46]. Liu et al. [44] found that the spontaneous activity of *MidFG_R* is significantly correlated with the severity of depression.

The *SPL* is a key region of the parietal lobe, known for its role in somatosensory and visuospatial integration, as well as its association with attention, written language, and working memory [47]. Limited prior research has investigated the connection between the parietal lobe and depression. However, this region is notably susceptible in PD patients. Previous research indicates that the parietal lobe is associated with cognitive function [48], frozen gait [49], apathy [50], tremor severity [51], and anxiety [28].

This study identifies a correlation between reduced fALFF values in the *MidFG.L* and *SPL.L* regions and depression in PD patients, suggesting their potential utility in diagnosing PD patients with depression. This indicates that the functional change of *MidFG.L* and *SPL.L* may signify depression in patients with PD.

Depression is marked by deficits in cognitive and emotional functions, as a regulator of the brain, *SN* playing a crucial role in sustaining both cognition and emotion in humans [52]. The *AC* and the *Putamen.R* are components of the *SN*. This study identified increased FC from the *AC* to the *MidFG.L*, as well as from the *Putamen.R* to *Cereb8.R*, *Cereb7.R*, and *IFG tri.L*. Lynch et al. [53] observed a duble expansion of the prestriatal *SN* network in the majority of depressed patients, and this expansion remained stable over time, regardless of fluctuations in mood state. The findings indicate that depression in PD patients is significantly influenced by changes in *SN* function.

The *SubCalC*, *aMTG.R*, and *AG.R* are all components of the *DMN*. This study showed that FC in these regions is modified in PD patients with depression. The *DMN* represents the organizational pattern of spontaneous neural activity in the brain and may be associated with learning, memory, mood, and cognitive functions [54]. Xu et al. [55] observed that dPD patients demonstrated reduced FC between the DMN and the auditory network in comparison to ndPD patients and healthy controls. Liao et al. [56] identified that the functional connectivity of the left hippocampus within the DMN and the right medial superior frontal gyrus within the *SN* were significant predictors of depression levels in PD. The results align with the previous finding that the *DMN* is a crucial network for altered function in nPD. In summary, *DMN* plays a significant role in depression among patients with Parkinson's disease.

This study presents several limitations: This research is a cross-sectional analysis and lacks long-term follow-up. Consequently, a more comprehensive longitudinal study is necessary in the future. The evaluation and categorization of depression relied solely on the HAMD- 17 scale, which introduces a level of subjectivity. Therefore, a comprehensive psychiatric assessment of patients is necessary. While LEDD employed partial correlation analysis to control for confounding variables, the potential impact of the medication on the results remains uncertain. This study only offers preliminary insights into the relationship between relevant indicators and depression in PD patients, necessitating further in-depth investigation.

## Conclusions

Our study identified a correlation between PD depression and serum SIRT3 levels, the total GMV/TIV ratios, the GMVs of *AMYG.R*, and fALFF values of the *MidFG.L* and *SPL.L* for the first time, suggesting possible marker for diagnostic depression in PD patients. Moreover, this study indicated that PD patients with depression show significant FC alterations in the *DMN* and *SN* when compared to those without depression. We expect that our findings will help clarify the biological and neural mechanisms underlying depression in PD patients.

## Data Availability

The raw data supporting the conclusions of this article will be made available by the authors without undue reservation.
